# Acute effects of isotonic eccentric exercise on the neuromuscular function of knee extensors vary according to the motor task: impact on muscle strength profiles, proprioception and balance

**DOI:** 10.3389/fspor.2023.1273152

**Published:** 2023-11-03

**Authors:** Carolina Vila-Chã, Antonio Bovolini, Cristiana Francisco, Ana R. Costa-Brito, Cláudia Vaz, María Rua-Alonso, José Antonio de Paz, Taian Vieira, Goncalo V. Mendonca

**Affiliations:** ^1^ Laboratory for the Assessment of Sports Performance, Physical Exercise and Health (Labmov), Polytechnic of Guarda, Guarda, Portugal; ^2^ Research Center in Sports Sciences, Health Sciences, and Human Development, Vila Real, Portugal; ^3^ Performance and Health Group, Department of Physical Education and Sport, Faculty of Sports Sciences and Physical Education, University of A Coruna, A Coruña, Spain; ^4^ Institute of Biomedicine, University of León, León, Spain; ^5^ Laboratorio di Ingegneria del Sistema Neuromuscolare (LISiN), Dipartimento di Elettronica e Telecomunicazioni, Politecnico di Torino, Turin, Italy; ^6^ PoliToBIOMed Lab, Politecnico di Torino, Turin, Italy; ^7^ Neuromuscular Research Laboratory, Faculdade de Motricidade Humana, Universidade de Lisboa, Lisbon, Portugal; ^8^ Interdisciplinary Center for the Study of Human Performance (CIPER), Faculdade de Motricidade Humana, Universidade de Lisboa, Lisbon, Portugal

**Keywords:** eccentric exercise, isotonic load, rate of force development, force steadiness, joint position sense, postural control

## Abstract

**Introduction:**

Eccentric exercise has often been reported to result in muscle damage, limiting the muscle potential to produce force. However, understanding whether these adverse consequences extend to a broader, functional level is of apparently less concern. In this study, we address this issue by investigating the acute and delayed effects of supramaximal isotonic eccentric exercise on neuromuscular function and motor performance of knee extensors during tasks involving a range of strength profiles, proprioception, and balance.

**Methods:**

Fifteen healthy volunteers (23.2 ± 2.9 years old) performed a unilateral isotonic eccentric exercise of the knee extensors of their dominant lower limb (4 × 10 reps at 120% of one Repetition Maximum (1RM)). The maximum voluntary isometric contraction (MVC), rate of force development (RFD), force steadiness of the knee extensors, as well as knee joint position sense and mediolateral (MLI) and anteroposterior stability (API) of the dominant lower limb, were measured pre-, immediately, and 24 h after the eccentric exercise. The EMG amplitude of the vastus medialis (VM) and biceps femoris (BF) were concomitantly evaluated.

**Results:**

MVC decreased by 17.9% immediately after exercise (*P *< 0.001) and remained reduced by 13.6% 24 h following exercise (*P *< 0.001). Maximum RFD decreased by 20.4% immediately after exercise (*P* < 0.001) and remained reduced by 15.5% at 24 h (*P *< 0.001). During the MVC, EMG amplitude of the VM increased immediately after exercise while decreasing during the RFD task. Both values returned to baseline 24 h after exercise. Compared to baseline, force steadiness during submaximal isometric tasks reduced immediately after exercise, and it was accompanied by an increase in the EMG amplitude of the VM. MLI and knee joint position sense were impaired immediately after isotonic eccentric exercise (*P *< 0.05). While MLI returned to baseline values 24 h later, the absolute error in the knee repositioning task did not.

**Discussion:**

Impairments in force production tasks, particularly during fast contractions and in the knee joint position sense, persisted 24 h after maximal isotonic eccentric training, revealing that neuromuscular functional outputs were affected by muscle fatigue and muscle damage. Conversely, force fluctuation and stability during the balance tasks were only affected by muscle fatigue since fully recovered was observed 24 h following isotonic eccentric exercise.

## 1. Introduction

In the last decades, eccentric exercise has gained a growing interest in sports and rehabilitation fields, leading to the development of a variety of training protocols through the manipulation of different training variables, including mechanical loads (1, 2). Eccentric exercise can be performed at constant angular velocity movement (isokinetic) or against a constant external load (isotonic), providing distinct mechanical loads as a stimulus, which might lead to specific neuromuscular adaptations. Most studies have used isokinetic mechanical loads to investigate the chronic and acute effects of eccentric training on different parameters of health and sports performance (3–5). However, isotonic loads are more commonly applied in the training field (2, 6). Isotonic eccentric training with external loads exceeding the load that subjects are able to lift for one repetition (1 RM load) has become popular among coaches, athletes, and fitness practitioners (6, 7). Several studies have shown that this training mode is more effective than isokinetic eccentric training in improving muscle strength, muscle architecture, and activation (5, 6, 8). The greater effectiveness of this type of training has been justified by the mechanical overload and the greater limb acceleration at the beginning of the isotonic contractions (6, 8). On the other hand, such mechanical stress can favor muscle damage and related delayed onset muscle soreness (DOMS) (3, 9, 10). Several studies have revealed that eccentric contractions are strongly associated with skeletal muscle impairments, consisting of structural disruption of sarcomeres, disturbed excitation–contraction coupling, and calcium signaling, leading to an inflammatory response (10–13). Moreover, these alterations are frequently accompanied by altered neural drive to the muscles (4, 14).

The neurophysiological changes induced by eccentric exercise can negatively impact neuromuscular function (3, 15, 16). Evidence has shown that isokinetic eccentric contractions of the knee extensors induce an acute and delayed decrease of different muscle force profiles, including maximal muscle strength (10, 13), rate of force development (4, 17), and force steadiness (18, 19). However, the magnitude of changes might vary depending on the muscle strength profile measured. Besides alterations in contractile properties, eccentric contractions can also disturb proprioception (20, 21). Significant disturbances in the joint position sense were found after maximal and sub-maximal eccentric exercise (22–24). Such impairments may contribute to an increased risk of injury and impaired postural control. Nevertheless, despite extensive research on the effects of eccentric exercise on proprioception, no study has investigated its impact on postural balance, especially when isotonic eccentric exercise with supramaximal loads is applied.

The extent to which eccentric exercise and related DOMS affects neuromuscular integrity and motor performance may vary depending on the mechanical characteristics of the eccentric load (5, 15). Moreover, the performance of different motor tasks seems to rely on distinct neuromuscular pathways and neural drive delivered to specific muscle groups. As a result, the impact of eccentric exercise and DOMS on motor performance can vary depending on the task specificity. Certain motor tasks may be more sensitive to the effects of DOMS, leading to a longer recovery period before optimal performance is restored. On the other hand, some motor tasks may exhibit resilience to DOMS or may recover more rapidly. Therefore, it is essential to consider the specific demands of different motor tasks when designing eccentric exercise programs and determining appropriate recovery strategies. Thus, this study aimed to investigate the acute effects of supramaximal isotonic eccentric exercise on different force profiles of the knee extensors, specifically maximum strength, rate of force development and force steadiness. Additionally, we aimed to evaluate the impact of this eccentric exercise mode on knee proprioception and dynamic balance. Changes in agonist and antagonist activity during force and balance tasks were also examined. By comprehensively investigating these factors, our study seeks to enhance the understanding of the implications of isotonic eccentric exercise and DOMS on neuromuscular function and motor performance. It was postulated that the magnitude of changes in the motor performance induced by a supramaximal isotonic eccentric exercise would depend on the motor task specificity. Moreover, we also hypothesized that changes in motor performance would be accompanied by alterations in the activation of the agonist and antagonist muscles.

## 2. Methods

### 2.1. Participants

Fifteen volunteers participated in the study (nine men and six women, age 23.2 ± 2.8 years; height 169.3 cm ± 6.5 cm; weight 63.8 ± 8.4 kg). All participants were practitioners of exercise, familiarized with strength training, and without any pathology in the dominant lower limb. The study was approved by the Polytechnic of Guarda Committee on Research Ethics and performed according to the Declaration of Helsinki. All subjects gave written informed consent before undertaking testing and training.

### 2.2. Experimental design

**Figure 1:**
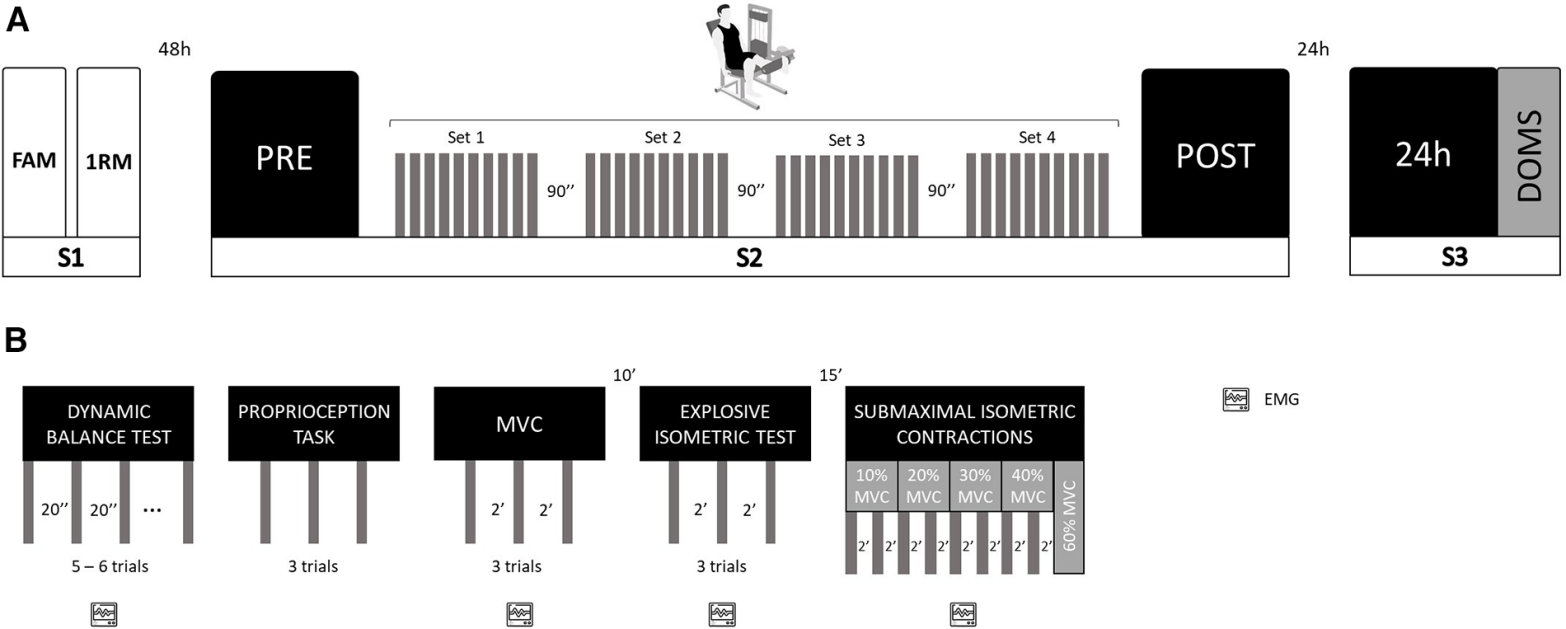
Schematical representation of the study design. (**A**) Experimental design; S, session; FAM, familiarization; 1RM, 1-repetition maximum test; PRE, assessments before isotonic eccentric protocol; POST, assessments immediately after isotonic eccentric protocol; 24 h, 24 h after isotonic eccentric protocol; DOMS, delayed onset muscle soreness; (**B**) Neuromuscular function assessment design; EMG, electromyographic recording; MVC, maximum voluntary isometric contraction.

The participants visited the laboratory 3 times: 48 h before the experimental sessions to get familiarized with the experimental setup and to estimate the 1RM of the knee extensor of the dominant lower limb (session 1); to perform the isotonic eccentric exercise session and assess the neuromuscular function before and immediately after exercise session (session 2) and 24 h after eccentric exercise for neuromuscular function reassessment and DOMS evaluation (session 3). All experimental measurements were assessed by following the same order: (i) dynamic balance; (ii) knee proprioception; (iii) maximum voluntary isometric contraction; (iv) explosive isometric contraction; and (v) force steadiness. The flow chart in Figure 1 summarizes the experimental protocol.

### 2.3. Isotonic eccentric exercise protocol

In order to determine the exercise intensity of the isotonic eccentric exercise, the 1RM of the knee extensors of the dominant lower limb was estimated for all participants using a multiple repetition test protocol and a regular leg extension machine (1MTH081, Panatta SLR, Italy). After a 5-min warm-up in a cycle ergometer, familiarization trials were performed to ensure proper execution of the test. The assessment protocol followed the guidelines outlined by National Strength and Conditioning Association Guidelines (25). The 1RM was estimated based on the Lombardi linear regression equation (26). The estimated 1RM was then used to define the exercise intensity of the isotonic eccentric protocol. After at least 48 h, the participants visited the laboratory to perform a single exercise session consisting of 4 sets of 10 repetitions at 120% of 1RM, with a recovery interval of 1 min and 30 s between sets. To guarantee proper technical execution and physical integrity of the participants, the following criteria were established: (i) participants were well seated in the machine, with their back well supported on the machine seat (support cushion adjusted individually); (ii) the participants were instructed to exert maximal force only during the descending phase of the movement (eccentric action) for 2 s; (iii) joint range of motion was established between 180° (complete extension) and 90° of knee flexion; (iv) the non-dominant lower limb remained stationary, in a neutral and supported position; and (v) the concentric phase of the movement was executed by two assistants.

### 2.4. Dynamic balance test

Unilateral dynamic balance of the dominant lower limb was measured with the Biodex Balance System (BBS) (model 945-300, United States of America). Participants were instructed to stand in their dominant lower limb on the BBS locked foot platform, keeping their body straight and the upper limbs lateral to the body and with the unsupported leg placed in a comfortable knee-flexed position without touching any surface. Then, the foot platform was released, and participants were asked to maintain an upright standing position for 20 s without contact with any other surfaces. During the test, the platform stability decreased progressively from level 8 to level 2. Each participant performed five to six trials with an interval in-between of 40 s. Deviations in the anteroposterior (x-axis) and mediolateral (y-axis) relative to the platform center were sampled at frequency of 20 Hz. Then, anteroposterior stability index (API), mediolateral stability index (MLI) and global stability index (SI) were computed with the BBS software, as described by Biodex Medical Systems, Inc. (27). API and MLI represent the variance of foot platform displacement in degrees for motion in the sagital and frontal plans, respectively (27). SI represents the variance of foot platform displacement in degrees, in all motions during a test. These indices were calculated using the degree of oscillation of the platform, in which low values indicated that the individual had good stability (28). For each participant the indexes corresponded to the average of the values obtained in the five to six trials. Electromyography (EMG) data of the vastus medialis (VM), and biceps femoris (BF) of the dominant leg were recorded while the participants were performing the test.

### 2.5. Knee proprioception test

The participants were seated in an elevated chair and blindfolded, with the knees approximately at 90° and the legs freely moving, without contact with any surface. Markers were placed on the external condyles of the femur (approximately 20 cm above the knee) and tibia (approximately 20 cm below the knee) for further knee angle position identification. The protocol consisted of positioning the non-dominant leg at an angle of 130° (manually measured with a goniometer), and subsequently participant was requested to move their dominant leg to match the position non-dominant leg as accurately as possible. Participants were allowed to practice the tasks and then repeated it three times. The trials were recorded with a video camera (Nikon, D3200, 1,920 × 1,080 pixels, 30 fps), positioned perpendicular to the subject, at two meters from the chair. The joint angle was then later assessed using the Kinovea program (version 0.8.15). For each trial the joint position error was assessed by computing: (*i*) absolute (shows the magnitude of the error); (*ii*) constant (indicates the direction of the error); and (*iii*) variable (indicates the response variability).

### 2.6. Muscle strength profiles

Maximal, explosive and submaximal isometric contractions of the knee extensors of the dominant lower limb were performed to assess the impact of the isotonic eccentric exercise on the muscle strength and motor control profiles. MVC was measured with the participants seated in an elevated chair (customized chair made by INEGI-UP), with their arms crossed in front of their chest and with the trunk and hips firmly strapped to the chair. The dominant leg was positioned at 90° of flexion and adjustable padded strap was placed around the tibiotarsal region, attached to a load cell (model load cell 614, SENSOR, United Kingdom), and fixed to the chair. The unassessed lower limb was positioned atop a box, ensuring it remained flexed, while exercising utmost caution to avoid applying any force. The participants were then instructed to progressively increase force against the padded strap and encouraged to exert their maximum within the first 5 s. Each participant performed 3 repetitions with 2 min of rest in between. The MVC value corresponded to the maximal force exerted in the three trials. Ten minutes after the MVC test, and in the same previous position, the participants performed three explosive isometric contractions, and they were encouraged to exert their maximum force as fast as possible. A rest of 2 min was given between trials.

After 15 min of rest, the participants performed submaximal isometric contractions at 10%, 20%, 30%, 40%, and 60% MVC, keeping it as stable as possible for 10 s. The participants completed two trials for each submaximal isometric contraction at 10%, 20%, 30%, and 40% MVC, as well as one trial for the submaximal isometric contraction at 60% MVC. A two-minute recovery interval was given between each trial. Participants were provided with visual feedback of the force exerted, that was displaced in computer screen of 22 inches in from them. It was required to the participants to maintain the target force level within two error bars of 2% MVC, centered around the target force level. EMG signals from the VM and BF and force were concomitantly record.

### 2.7. Assessment of muscle pain

To confirm the presence of DOMS 24 h post-eccentric exercise, participants verbally rated their perceived pain on a scale from 0 (“‘no soreness”') to 10 (“‘worst soreness imaginable”'). The subjects were asked to rate the average pain intensity in the quadriceps during their regular activities of daily living (e.g., descending stairs) since their last visit to the laboratory (over the past 24 h).

### 2.8. EMG and force recordings

Surface EMG signals were acquired from the VM and BF muscles during the dynamic balance and isometric tasks used to measure the different muscle strength profiles. Signals from the selected muscles were recorded with three pairs Ag-AgCl electrodes (Ambu Neuroline, Denmark; conductive area 28 mm^2^), placed as recommended by Hermens et al. (29). Before placement of the electrodes, the skin was shaved, lightly abraded, and cleansed with water. A ground electrode was placed around the ankle of the dominant lower limb. Surface EMG signals were amplified as bipolar derivations (EMG amplifier, OT Bioelettronica, Turin, Italy), band-pass filtered (−3 dB bandwidth, 10–500 Hz), sampled at 2048 samples/s, and converted to digital data by a 12-bit A/D converter board. The EMG electrodes remained in place during the exercise session and were used for all EMG measurements on the same day (before and immediately after training). At the end of the experiment, the electrodes were removed, and the electrode positions were marked on the skin to ensure that the electrodes were placed in the same location for the EMG recordings obtained 24 h after training. The force signals were measured with a load cell (load sensitivity = 0.0048 V/N; SENSOR, load cell 614, United Kingdom) and collected through an auxiliary channel of the EMG amplifier (OT Bioelettronica, Italy), sampled at 2048 samples/s, and converted to digital data by a 12-bit A/D converter board and recorded in the OT Biolab software (OT Bioelettronica, Italy), thus allowing the simultaneous collection of force and EMG signals.

### 2.9. EMG and force signal analysis

For the reference MVC, the average rectified value (ARV) was computed from a time interval of 250 ms centered at instant of the maximal force. During the explosive contractions, the ARV was computed from two-time intervals: (i) 70 ms prior to the onset of the contraction and (ii) 50 ms centered at the time instant of the maximal slope (19). The ARV values for each muscle and task were normalized by the respective muscle's ARV obtained during the MVC [(task ARV/ARV MVC) × 100]. During dynamic balance tests the percent of activation time of the VM and BF was determined by using the algorithm described by Vincent and Soile (30). The analog signals from the load cell were converted into force (N, Newton) and the maximum rate of force development (RFD) was computed as the maximum slope of the force-time curve. The onset of the contraction was defined as the time instant that force exceeded 3 times the standard deviation value observed at baseline. For each submaximal contraction, the coefficient of variation (CoV) of force was calculated over the entire duration of the contraction. The CoV of force was computed by dividing the standard deviation (SD) of the force signal by the mean force multiplied by 100 [(SD/mean force) × 100]. This measure expresses the absolute force variability as a fraction of the mean force exerted (31).

### 2.10. Statistical analysis

Statistical analyses were performed using the Statistical Package for Social Sciences (SPSS Version 24, IBM Corporation, Armonk, New York, USA) software. The normality of the dependent variables was confirmed using the Shapiro-Wilk test. The acute effects of the isotonic eccentric training on muscle force profile (MVC and maximum RFD), dynamic balance (GBI, API and MLI), proprioception (absolute, constant and variable errors) and muscle activity were assessed with one-way repeated-measures ANOVA, with factor time (Pre-, Post-, and 24 h after isotonic eccentric exercise). During submaximal isometric contractions, changes in the CoV of the force and ARV of the VM and BF were assessed with a two-way repeated measures ANOVA with factor time and target force levels (10%, 20%, 30%, 40% and 60% of MVC). Pairwise comparisons were performed with the Bonferroni *post hoc* test when ANOVA was significant. Partial eta-squared (ŋp^2^) was used to calculate the effect size of the statistical results, which were classified as weak (ŋp^2 ^< 0.01), medium (ŋp^2^ 0.01 < 0.06) or high (ŋp^2 ^> 0.14) (32). The significance level was set to *P* < 0.05. Results are reported as means ± SD.

## 3. Results

### 3.1. Pain perception

Twenty-four hours after isotonic eccentric exercise, the participants rated their perceived pain intensity as 3.6 ± 2.6 on a scale from 0 to 10.

### 3.2. Maximum voluntary contraction

**Figure 2:**
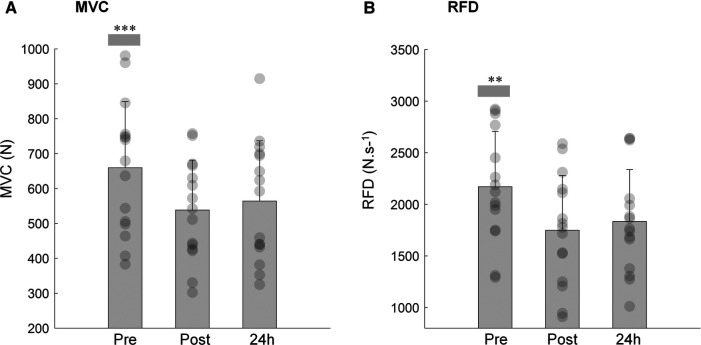
Mean and SD of the maximal voluntary contraction (**A**; MVC) and rate of force development (**B**; RFD) of the knee extensor pre, post and 24 h after isotonic eccentric exercise. ***MVC immediately post and 24 h after exercise was significantly reduced when compared with pre-condition (*P *< 0.001). **RFD immediately post and 24 h after exercise was significantly reduced when compared with pre-condition (*P *< 0.001).

The MVC of the knee extensors decreased immediately after (−17.9%; *P *< 0.001) and 24 h after training session (−13.6%; *P* < 0.001) compared to pre-exercise values (Figure 2A; main effect: *P *< 0.001, ŋp^2^ = 0.671). These alterations were accompanied by a decline in the ARV of the vastus medialis (*P *< 0.040, ŋp^2^ = 0.238; Table 1), while no changes were observed in the ARV of the biceps femoris (*P *< 0.207, ŋp^2^ = 0.106; Table 1). Following exercise session, the vastus medialis ARV significantly decreased (*P *= 0.017) returning to baseline values 24 h after (*P *= 1.000).

**Table 1:** Average rectified value (ARV) for the vastus medialis (VM) and biceps femoris (BF) obtained during the maximal voluntary contraction (MVC), the isometric explosive contractions at onset of the contraction and at time instant that maximum rate of force development (RFD) was reached and during submaximal isometric contractions at target force levels of 10%, 20%, 30%, 40% and 60% MVC obtained at baseline (pre), immediately post, and 24 h after isotonic eccentric exercise.

	Vastus medialis			Biceps femoris		
Variable	Pre	Post	24 h	Pre	Post	24 h
MVC [ARV (µV)]	367.72 ± 122.65	312.66 ± 110.25^§^	347.92 ± 130.38	113.04 ± 63.50	120.61 ± 45.01	97.28 ± 25.21
RFD onset (% ARV MVC)	59. 63 ± 23.70	79.80 ± 20.27^§^	62. 99 ± 24.92	71.99 ± 25.74	86.23 ± 34.92	76.70 ± 36.34
RFD max (% ARV MVC)	69.89 ± 15.33	92.50 ± 33.49^‖^	72.58 ± 33.49	69.78 ± 13.05	82. 82 ± 24.88	80. 01 ± 26.23
10% MVC (% ARV MVC)	12.53 ± 3.64	16.22 ± 5.29^§,†^	11.93 ± 3.97	14.42 ± 4.52	17.13 ± 8.31	15.97 ± 5.25
20% MVC (% ARV MVC)	21.62 ± 6.24	29.59 ± 8.86^§,‡^	20.52 ± 6.37	22.51 ± 4.95	28.27 ± 10.31	25.04 ± 7.84
30% MVC (% ARV MVC)	33.06 ± 6.93	41.50 ± 9.72^‡,†^	32.89 ± 10.59	35.30 ± 7.03	39.01 ± 12.43	36.92 ± 13.48
40% MVC (% ARV MVC)	46.03 ± 11.20	56.39 ± 10.68^§,‖^	45.98 ± 12.05	50.39 ± 16.05	39.32 ± 8.11	41. 91 ± 10.39
60% MVC (% ARV MVC)	48.97 ± 8.84	53.83 ± 7.65	49.62 ± 11.97	39. 78 ± 8.11	41. 91 ± 10.39	39.78 ± 11.27
Dynamic balance _(% time activation)_	46.62 ± 15.53	57.17 ± 13.86	49.82 ± 23.10	38.83 ± 16.13	45.67 ± 15.88	39.73 ± 21.80

^†^ Post significantly different from 24 h (*P* < 0.01).

^‡^ Post significantly different from pre-condition (*P* < 0.01).

^§^ Post significantly different from pre-condition (*P* < 0.05).

^‖^ Post significantly different from pre and after 24 h (*P* < 0.05).

### 3.3. Rate of force development

As for MVC, the maximum RFD was substantially reduced immediately after the isotonic eccentric exercise (−20.3%; *P *< 0.001), remaining diminished 24 h following exercise (−15.5%; *P *< 0.001) when compared to pre-exercise values (Figure 2B; time effect: *P *< 0.001, ŋp^2^ = 0.608). The time to reach maximum RFD did not differ between pre (179 ± 3.6 ms), post (183 ± 4.0 ms) and 24 h after eccentric exercise (174 ± 2.9 ms) (time effect: *P *= 0.615.) The EMG amplitude of the VM, at onset of the explosive isometric contraction (time effect: *P* = 0.025, ŋp^2^ = 0.435) and at the time instant that maximum RFD was reached (time effect: *P* = 0.014, ŋp^2^ = 0.483) increased immediately post (*P* < 0.05, for both time intervals) and returned to baseline 24 h after exercise session (Table 1). No alterations were observed in the ARV of the BF for both time intervals of the explosive contractions (Table 1, time effect: *P* > 0.180, ŋp^2^ > 0.122, in both time intervals).

### 3.4. Force steadiness

**Figure 3:**
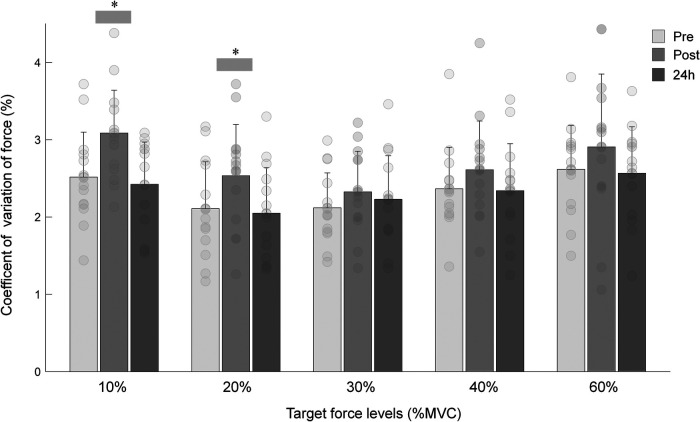
Mean and SD of the coefficient of variation (CoV) of force during submaximal isometric contractions at target force levels of 10%, 20%, 30%, 40% and 60% MVC obtained at baseline (pre), immediately post, and 24 h after eccentric exercise. *Indicates that CoV post eccentric exercise is significantly different from the CoV measured before (pre) and 24 h after exercise (*P *< 0.05).

Figure 3 shows the CoV of force at the different targe force levels measured pre, immediately post and 24 h after isotonic eccentric exercise. Data analysis revealed a significant effect for both factors load (*P *= 0.004, ŋp^2^ = 0.289) and time (*P *< 0.001, ŋp^2^ = 0.491). CoV at 10% MVC was higher than the CoV observed at 20% and 30% of MVC (*P *= 0.028 and *P* = 0.042, respectively). At 60% MVC, there was a significantly higher CoV compared to the CoV at 20% and 30% (*P* = 0.020 and *P* = 0.013, respectively).

The pooled data showed that CoV significantly increased post exercise (+14.7%; *P* = 0.025), returning to baseline values in the 24 h after (Figure 3). No interaction time × load was observed (*P* = 0.343, ŋp^2^ = 0.075). When stratified by force levels, the results revealed that only at 10% and 20% MVC, the CoV was significantly higher immediately after exercise (*P* = 0.014), returning to baseline values 24 h after exercise. Changes in the force steadiness were accompanied by significant alterations in the ARV of the VM (time × load effect: *P* = 0.041, ŋp^2^ = 0.166; Table 1). The ARV of the VM increased immediately after exercise for at all force levels (*P* < 0.030), except for the 60%MVC (*P* = 0.331). Twenty-four hours later the ARV values returned to baseline (Table 1). No significant changes were observed in the EMG amplitude of the biceps femoris either immediately post or 24 h after the eccentric exercise (time effect: *P* = 0.441, ŋp^2^ = 0.057; Table 1).

### 3.5. Proprioception

**Figure 4:**
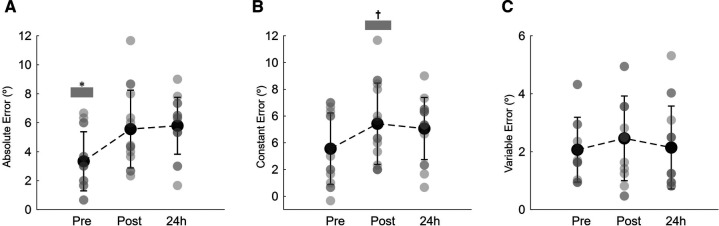
Mean and SD of the absolute joint position error (**A**), constant error (**B**) and variable error (**C**) during the knee repositioning task recorded at baseline (pre), immediately post and 24 h post eccentric exercise. *Indicates that absolute joint position error pre-eccentric exercise significantly lower than immediate post and 24 h after eccentric exercise (*P *< 0.05). ^†^Denotes a significant difference between post and pre constant error values (*P *< 0.05).

Figure 4 illustrates the absolute, constant, and variable errors during the knee repositioning task, before, immediately post, and 24 h after the isotonic eccentric session. The results showed that participants significantly increased the magnitude of the repositioning error following eccentric exercise (time effect: *P *= 0.002 ŋp^2^ = 0.358; Figure 4A). The absolute error increased immediately after exercise (+2.4 ± 2.1°; *P* = 0.021) and remained higher 24 h after when compared to baseline (+2.7 ± 2.7°; *P* = 0.020) (Figure 4A). After training, participants moved into a more extended knee position relative to the reference leg, as indicated by the constant error (time effect: *P *= 0.012 ŋp^2^ = 0.270; Figure 4B). Immediately after the training session, the constant error increased by +5.5 ± 3.1° when compared to baseline (*P* = 0.015). Twenty-four hours later the constant error still increased, but no statistical differences were observed between pre and 24 h after training conditions (*P* = 0.133; Figure 4B). The position consistency, as indicated by the variable error, although declined was not significantly affected by the present exercise protocol (time effect: *P *= 0.435 ŋp^2^ = 0.057; Figure 4C).

### 3.6. Dynamic balance

**Figure 5:**
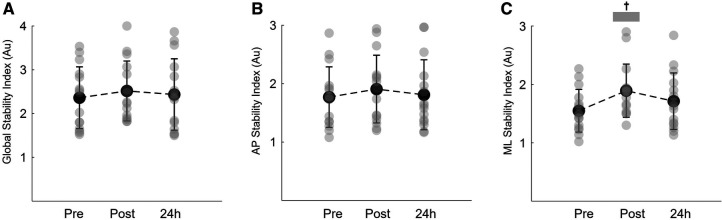
Mean and SD of the global stability index (**A**) anteroposterior stability (AP) index (**B**) and mediolateral stability (ML) index (**C**) during the unilateral dynamic balance task recorded at baseline (pre), immediately post and 24 h post eccentric exercise. ^†^Denotes a significant difference between post and pre constant error values (*P *< 0.001).

Figure 5 shows the results on the global balance, anteroposterior and mediolateral stability indexes before, immediately post and 24 h after eccentric isotonic exercise. Global balance and anteroposterior indexes were not affect by the exercise protocol (time effect: *P *= 0.483 ŋp^2^ = 0.051 and *P *= 0.685 ŋp^2^ = 0.025, respectively; Figures 5A,B). Nonetheless, significant alterations were observed in the mediolateral direction (time effect: *P *= 0.002, ŋp^2^ = 0.399; Figure 5C). The instability in the mediolateral direction significantly increased immediately post exercise (*P* < 0.001). Following 24 h, the mediolateral index remained higher, but no statistical differences were observed when compared to baseline values (*P* = 0.092) (Figure 5C).

Although there was an increase in the relative time of VM and BF activation during balance tests immediately after the eccentric training, the results were not statistically significant (time effect: *P* > 0.434; ŋp^2^ < 0.080, for both muscles; Table 1).

## 4. Discussion

The present study showed that eccentric exercise with a supramaximal isotonic load caused DOMS and impaired motor output across different motor tasks. Force output assessed either by MVC, RFD or force steadiness declined immediately after eccentric training. Twenty-four hours later, MVC and RFD remained impaired, while isometric force steadiness, at different target levels, was recovered. These alterations were accompanied by adjustments in agonist muscle activity that followed a different time course and pattern depending on the motor task characteristics. Postural control and proprioception were also impaired by the isotonic eccentric exercise as well. The stability in the mediolateral direction decreased, and the error magnitude of knee positioning increased immediately after exercise and in the presence of DOMS. Collectively, these findings demonstrate that the magnitude and the time course of the impairments induced by eccentric exercise with a supramaximal isotonic vary depending on the motor task specificity.

### 4.1. Muscle soreness and muscle strength performance

The participants reported soreness in the quadriceps 24 h after training, confirming the presence of DOMS. The average soreness level was 3.6 out of 10, which is in agreement with studies on acute responses to isokinetic eccentric exercise (3, 19, 33). Both MVC and RFD of the knee extensors decreased immediately after isotonic eccentric exercise and remained impaired 24 h later. Nonetheless, the magnitude of decline in the RFD post and 24 h after exercise was greater than in the MVC (20.3% and 15.5% vs. 17.9% and 13.6%, respectively). Several studies have reported similar results, suggesting that RFD is more sensitive to muscle fatigue (34, 35) and muscle damage after eccentric exercise (15, 17, 19, 36). The observed decrease in force-generating capacity is a well-known consequence of performing unaccustomed and/or vigorous eccentric exercise. This type of exercise can lead to disruptions in cytoskeletal structures and microlesions in muscle fibers, thereby affecting the excitation-contraction coupling mechanisms (16, 37). Consequently, a long-lasting deficit in force production (> 24 h) is commonly observed (38, 39). The magnitude of the force production deficit and time course of the recovery seems to be partially determined by the content and size of the muscle fibers type II (4). Several studies have shown that type II fibers are particularly susceptible to damage from intense eccentric exercise, mainly due to their higher tension generating capacity and shorter optimum length for tension (10, 40). Although we did not directly measure muscle fiber damage, it is very likely that the greater changes observed in the RFD are a consequence of subcellular perturbances within type II fibers. Fiber type is often considered a major factor influencing RFD, in which type II fibers play an important role in producing faster rates of tension development (9). Muscle activation is also a critical determinant of RFD, particularly in the earlier phases of the rising force during rapid contractions (41). Given the potential for muscle damage caused by eccentric exercise, neural adaptations may occur as an effort to overcome muscle impairments and generate the required muscle tension. In the current study, the EMG amplitude of the vastus medialis exhibited a significant increase at the onset of rapid contraction and around the time of maximum RFD immediately after eccentric exercise, while no changes were observed in the antagonist activity. Still, EMG amplitude of the vastus medialis returned to baseline levels within 24 h, even though RFD impairment persisted.

The findings indicate that changes in EMG patterns are strongly associated with neuromuscular fatigue, rather than the neuromuscular mechanisms that cause maximum RDF impairments from isotonic eccentric exercise. While several factors could potentially account for the immediate post-eccentric exercise increment in the EMG amplitude, it is highly plausible that these changes are a consequence of an increment of the recruitment and/or firing rate of the motor units (18, 42). Because at the contraction onset and at the instant of maximum RFD, force is submaximal, the neural drive can be increased to compensate the force impairments induced by muscle fatigue. In fact, increased EMG amplitude of the agonist muscle was also observed during the sustained submaximal contractions immediately post, but not in the 24 h after eccentric exercise. The presence of muscle fatigue is also indirectly confirmed by the decline in EMG activity during the MVC. Contrary to submaximal efforts, during maximal contractions, the EMG amplitude typically decreases in the presence of muscle fatigue, indicating the limitations of the central nervous system to continually increase synaptic input to the motoneurons (43).

Peñailillo et al. (17) did not observed any change in the EMG amplitude of the vastus lateralis during explosive contractions following eccentric cycling protocols at 60% of maximal concentric contraction. Conversely, Vila-Chã et al. (19) reported a decline of the EMG amplitude of the vasti muscles at the contraction onset and at the time of maximum RFD, which persisted in the 24 h after a maximal isokinetic eccentric exercise protocol. The conflicting results might be explained by differences arising from variations in the modes of eccentric exercise employed across each study, that might have induced different magnitudes of fatigue and muscle damage. It is well known that both phenomena are dependent of modulation based on task-specific factors, which in turn govern the neurophysiological changes (44, 45). In the current study, the immediate decline in the maximum RFD and MVC after isotonic eccentric exercise is very likely explained by a combination of fatigue and initial muscle damage, leading to changes in muscle activation. However, the sustained alteration in RFD and MVC values observed 24 h after isotonic eccentric exercise appears to be predominantly stem from peripheral adaptations prompted by muscle damage, with negligible impact on the neural drive.

Another important parameter about muscle strength is the force steadiness measured during submaximal contractions. It has been shown that force steadiness during submaximal isometric contractions is moderately associated with performance on different motor tasks, such as standing balance and walking performance (31). Therefore, gaining insight into the influence of isotonic eccentric exercise on force fluctuation can yield valuable functional and physiological information regarding complementary indicators of function recovery after eccentric exercise. Such significance is accentuated by the fact that this parameter is predominantly influenced by neural factors that are not directly associated with MVC or RFD. The present study showed that force steadiness during different submaximal isometric levels significantly decreased immediately after eccentric exercise, but returned to baseline values in the 24 h after exercise. The EMG amplitude of the VM followed a similar pattern and no changes were observed in the activity of the antagonist muscle. The results are in line with previous studies that applied isokinetic eccentric protocols (19, 23, 46). In these studies, the force steadiness of the elbow flexors (23, 46) and knee extensors (19) decreased immediately after eccentric exercise for all submaximal levels (between 2.5% and 50% MVC). Twenty-four hours later the force steadiness remained impaired only during the submaximal contractions at very low intensities (≤ 10% MVC). The observed alterations were accompanied by an increased EMG amplitude of the agonist muscles, indicating the presence of low-frequency fatigue following eccentric exercise (46, 47). In the current study, the lowest intensity used was 10% MVC. Even for this very low intensity, the force steadiness of the knee extensors was recovered 24 h after the eccentric exercise. The findings suggest that the CoV of force is a better indicator of muscle fatigue than potential muscle damage resulting from the isotonic eccentric protocol applied in this study.

### 4.2. Proprioception and balance

Immediately after isotonic eccentric exercise, an increase of the position errors was observed. The magnitude of the error (measured as absolute error) remained higher after 24 h after exercise, while direction of the error (constant error) and position consistency (variable error) returned to pre-exercise values. The results indicate that the participants matched the predetermined knee position by adopting a more extended position. Similar results have been obtained previously (20–22, 48–50). It is well known that a period of intense exercise increases errors in limb position sense (51). The disturbance in joint position sense seems to arise from the altered sense of effort due to the presence of fatigue, either after concentric or eccentric exercise (24, 52). Moreover, exercise effects on position sense are directly dependent on the level of muscle fatigue produced by the exercise (51). It has been shown that the accumulation of metabolites, stimulation of small muscle afferents in the group III and IV due to muscle fatigue might be involved in the sense of effort, perturbing the sense of position (51, 53). However, following eccentric exercise it was observed that position matching errors might persist in the subsequent days, where chemical products of fatigue were long gone (21, 24, 50). This suggests that factors beyond fatigue affected the position sense. In the past it has been hypothesized that eccentric exercise would cause also damage to muscle spindles, leading to perturbations in proprioception. Nonetheless, animal studies have shown that after intense eccentric exercise the responsiveness of muscle spindles is not disturbed despite extensive muscle damage (51, 54).

Tsay et al. (50) observed an association between de decrease in force and increase position errors, suggesting that exercise-induced decline in force (of 20%–30%) can disrupt limb position sense. It has been suggested that the effects of exercise on proprioception resides on the operation of an internal feed-forward model (53, 55). This hypothesis indicates that the expected sensory feedback for a particular limb position is based on past memories and compares it with the actual sensory feedback from the fatigued limb. Based on previous experience, the sensory feedback from the fatigued muscle might be greater than anticipated from the motor command, altering the neural drive to the fatigued limb (23, 50). Conversely Da Silva et al. (48) observed a better association between position matching errors and voluntary activation deficits than with MVC decline. However, in their study, and despite a long-lasting MVC decrease and the presence of DOMS up to 48 h postexercise, position errors returned to baseline values within in the 24 h after eccentric exercise. In line with Tsay et al. (50) results, in the present study, the magnitude of errors increased immediately after eccentric exercise and remained higher 24 h later, which accompanied the observed changes in MVC and RFD.

Notwithstanding the observed changes in position sense, during the balance task only alterations in the mediolateral stability index were observed. This parameter was increased immediately after isotonic eccentric exercise but returned to pre-exercise values 24 h after. The global and the anteroposterior stability index was not affected by the exercise protocol. Dabbs & Chander (56) investigated the effects of eccentric exercise on balance performance and despite the significant reductions in knee extensor torque up to at least 48 h, no changes were observed during the balance task. The lower sensitivity of the balance task to fatigue and muscle damage induced by the eccentric exercise might be explained by the fact that postural control is not only dependent of the proprioceptive and somatosensory system (57). The maintenance of upright balance is a complex task that involves afferent visual and vestibular systems that are not primary affected by muscle fatigue or muscle damage (58).

Although this study provides insights into the complex effects of supramaximal isotonic eccentric exercise on neuromuscular function, motor performance, and proprioception, it is essential to acknowledge its specific limitations. Firstly, muscle damage induced by the isokinetic eccentric exercise was not directly measured, enabling further investigation of the relationship between muscle damage characteristics and the degree of motor performance impairments across a variety of motor tasks. The study focused on the 24 hours after exercise recovery period. However, extending the follow-up duration could shed light on the longer-term effects and the persistence of neuromuscular alterations induced by eccentric exercise. The evaluation of postural control during a balance task revealed a degree of insensitivity to fatigue and muscle damage in this study. To provide a more comprehensive understanding on the impact of the eccentric exercise on balance, a thorough analysis of additional balance-related parameters would be necessary. Lastly, given the highly specific nature of the eccentric exercise protocol used in this study, it is important to acknowledge that the results are inherently constrained by the confines of the defined experimental protocol. Consequently, the applicability of these findings to broader eccentric exercise regimens or different populations may be limited.

## 5. Conclusion

The supramaximal isotonic eccentric exercise affected differently the performed motor tasks. When compared to baseline, both, MVC and RFD were impaired immediately after exercise and in the subsequent 24 h. However, the magnitude of changes was more pronounced in the RFD than in the MVC knee extensors profile. Proprioception of knee position was also affected by the isotonic eccentric exercise and remained altered 24 hours after the exercise protocol. The results revealed that both RFD, MVC and position sense are affected by muscle fatigue and muscle damage. Conversely, force steadiness during submaximal contractions and mediolateral stability index during the balance task were only impaired immediately after eccentric exercise, returning to baseline values within 24 h. The current study showed that isotonic eccentric exercise induces specific alterations on different neuromuscular functional outputs. The findings underscore the importance of understanding the specific alterations and recovery timelines associated with eccentric exercise for effective exercise and rehabilitation planning. However, future research needs to be performed to enhance understanding of these phenomena.

## Data availability statement

The raw data supporting the conclusions of this article will be made available by the authors, without undue reservation, under request.

## Ethics statement

The studies involving humans were approved by Polytechnic of Guarda Committee on Research Ethics. The studies were conducted in accordance with the local legislation and institutional requirements. The participants provided their written informed consent to participate in this study.
